# Prevalence and correlates of problematic smartphone use in a large random sample of Chinese undergraduates

**DOI:** 10.1186/s12888-016-1083-3

**Published:** 2016-11-17

**Authors:** Jiang Long, Tie-Qiao Liu, Yan-Hui Liao, Chang Qi, Hao-Yu He, Shu-Bao Chen, Joël Billieux

**Affiliations:** 1 Mental Health Institute of the Second Xiangya Hospital, Central South University, The China National Clinical Research Center for Mental Health Disorders, National Technology Institute of Psychiatry, Key Laboratory of Psychiatry and Mental Health of Hunan Province, Changsha, Hunan People’s Republic of China; 2 Laboratory for Experimental Psychopathology, Psychological Science Research Institute, Université Catholique de Louvain, Louvain-la-Neuve, Belgium; 3 Internet and Gambling Disorders Clinic, Department of Adult Psychiatry, Cliniques Universitaires Saint-Luc, Brussels, Belgium; 4 Department of Psychiatry and Biobehavioral Sciences and Semel Institution for Neuroscience and Human Behavior, David Geffen School of Medicine, University of California, California, USA

**Keywords:** Smartphone addiction, Problematic smartphone use, Perfectionism, Prediction, Perceived stress, Mobile phone addiction, Mobile phone problematic use, Risk factors

## Abstract

**Background:**

Smartphones are becoming a daily necessity for most undergraduates in Mainland China. Because the present scenario of problematic smartphone use (PSU) is largely unexplored, in the current study we aimed to estimate the prevalence of PSU and to screen suitable predictors for PSU among Chinese undergraduates in the framework of the stress-coping theory.

**Methods:**

A sample of 1062 undergraduate smartphone users was recruited by means of the stratified cluster random sampling strategy between April and May 2015. The Problematic Cellular Phone Use Questionnaire was used to identify PSU. We evaluated five candidate risk factors for PSU by using logistic regression analysis while controlling for demographic characteristics and specific features of smartphone use.

**Results:**

The prevalence of PSU among Chinese undergraduates was estimated to be 21.3%. The risk factors for PSU were majoring in the humanities, high monthly income from the family (≥1500 RMB), serious emotional symptoms, high perceived stress, and perfectionism-related factors (high doubts about actions, high parental expectations).

**Conclusions:**

PSU among undergraduates appears to be ubiquitous and thus constitutes a public health issue in Mainland China. Although further longitudinal studies are required to test whether PSU is a transient phenomenon or a chronic and progressive condition, our study successfully identified socio-demographic and psychological risk factors for PSU. These results, obtained from a random and thus representative sample of undergraduates, opens up new avenues in terms of prevention and regulation policies.

**Electronic supplementary material:**

The online version of this article (doi:10.1186/s12888-016-1083-3) contains supplementary material, which is available to authorized users.

## Background

Because of its extremely large population base, Mainland China has the most mobile phone users worldwide. In January 2016, the Ministry of Industry and Information Technology of China announced that over 1.3 billion Chinese people (95.5%) have their own mobile phones [1]. As smartphones emerged in recent years, China also became the largest consumer market of smartphones, advancing at an astonishing pace. According to the International Data Corporation, in 2014 alone, more than 420 million smartphones were sold in Mainland China [2].

Smartphones no longer need to be considered simply as “mobile phones.” With various enhanced functions, a smartphone is not just a communication tool, but a real-time information provider and a powerful portable computer. Although the smartphone brings conveniences to people’s daily lives, it is also associated in certain cases with patterns of addictive usage involving negative outcomes. According to Billieux and colleagues [3, 4], problematic smartphone use (PSU) is defined as “an inability to regulate one’s use of the smartphone, which eventually involves negative consequences in daily life.” Several studies have introduced the term “smartphone addiction” on the basis of similarities in symptoms displayed by excessive smartphone users and substance abusers (e.g., loss of control, cognitive salience, mood regulation) [5, 6]. Nevertheless, to date, the evidence that problematic (smart)phone use constitutes a genuine addiction is scarce, especially regarding issues such as tolerance and withdrawal [4]. To reduce the risk of pathologization [7], we decided to use the term *problematic smartphone use* (PSU) to describe the condition. Existing studies reported that problematic mobile phone use was associated with various worrisome physical and psychological issues [8–13], which suggests that excessive usage may also bring about adverse effects in the realms of psychological well-being, interpersonal relationships, and physical health. Moreover, as smartphones are much more complex and enhanced than traditional mobile phones, Kim et al. [14] argued that the adverse effects caused by PSU might be even more exacerbated than they are with mobile phones.

Compared with older social groups, undergraduates were shown to be more vulnerable to PSU [15, 16]. As young people, undergraduates are highly interested in smartphones, yet the critical transition of their psychological and cerebral development is not yet completed. Moreover, today’s undergraduates are digital natives who have grown up surrounded by (smart)phones and integrated this instrument into part of their lifestyle and identity [17]. Tao et al. [18] assessed problematic mobile phone use with the Self-rating Questionnaire for Adolescent Problematic Mobile Phone Use in a large random sample of Chinese middle school students and high school students in 2012, and the prevalence of problematic mobile phone use among Chinese adolescents was estimated to be 26.2%. However, limited data are available in the literature about the prevalence of PSU by using a reliable sampling method with a sufficiently large sample of Chinese undergraduates.

The current landscape of smartphone use among Chinese undergraduates may be different from other countries and regions of the world. Indeed, it has been established that socio-environmental and cultural factors influence the nature and type of excessive and addictive behaviors displayed in a specific country or geographic area [19–21]. As a developing country with a huge population, China has its cultural particularity and special practices. Moreover, because the proliferation of smartphones and the development of the Internet in China have been incredibly fast, this macro-level transition may have an impact on undergraduate students as well. Although PSU might be a serious problem for Chinese undergraduates, few studies to date have explored this topic in Mainland China.

### Correlates of PSU

The etiology and underlying processes of PSU remain unclear. According to Billieux et al. [3, 7], most existing studies on this topic were conducted without a defined theoretical framework, which might limit the possibility for a better understanding of PSU. Nonetheless, a growing number of studies have explored the correlates of PSU and identified various socio-demographic and psychological risk factors.

### Socio-demographics

Previous studies emphasized associations between specific socio-demographic variables and PSU. Among these studies, several identified a gender effect, with females reporting more intensive use and symptoms of addictive use than males [22–25]. Regarding age, some studies reported that younger individuals are more likely to show elevated use and symptoms of dependence on the mobile phone than are older individuals [15, 16]. Moreover, socioeconomic status [26, 27] was also related to addictive mobile phone use, although the results were inconsistent. Some studies suggested that lower socioeconomic status is related to problematic mobile phone use [26, 27], whereas other studies pointed to the opposite conclusion [27–29]. One study also found that students majoring in humanities were more likely to use mobile phones more problematically than were those majoring in natural science [30]. Given these previous findings and taking into account the Chinese context of college education, we expected that females, junior undergraduates, and those majoring in humanities or with immoderate monthly income from the family would be more at risk for PSU.

### Psychological factors

Influential models have proposed that excessive and addictive behaviors (e.g., substance abuse, deregulated sexual and eating behaviors, problematic gambling, and problematic video gaming) are displayed to reduce or alleviate aversive states through temporary cognitive and/or behavioral escape [19, 31, 32]. Therefore, addictive-like behaviors could be considered as consequences of distraction, and avoidance-based strategies could be used to manage chronic stressors and aversive emotions. Such a hypothesis is also indirectly supported by the many studies that have linked problematic (smart)phone use with personality traits that act as risk factors for addictions, including neuroticism [33, 34] and impulsivity [3, 15, 23, 35–37]. Accordingly, several potential risk factors emerged from a stress-coping approach to PSU, such as psychopathological symptoms (anxiety, depression) and perceived stress.

Experience of aversive emotions and negative mood states are core predictive candidates for PSU according to the stress-coping theory. Previous research consistently reported associations between psychopathological symptoms such as anxiety and depression and various addictive and excessive behaviors, including alcohol abuse [38], smoking [39], and Internet-related disorders [40, 41]. Several studies also highlighted that depression and anxiety symptoms were similarly associated with problematic mobile phone use [5, 42–44], leading scholars to hypothesize that smartphone use can serve to relieve negative effect in depression- or anxiety-prone individuals and thus produce addictive patterns of use [42, 45]. In the present study, we expected that both depression and anxiety would be positively associated with PSU.

Elevated perceived stress occurs when individuals are faced with a situation appraised as demanding and/or threatening while insufficient resources are available to cope with this situation [46]. Several recent studies reported that subjective stress was positively associated with addictive smartphone use [6, 47]. Consistent with the stress-coping framework described earlier, Chiu [6] proposed that smartphone might be used as a distraction from stressful experiences and thus act as coping mechanism. From the existing studies, we expected that perceived stress would be positively associated with PSU.

An eventual potential risk factor for PSU, which has to our knowledge not yet been investigated, is perfectionism, defined as a person’s striving for flawlessness and setting excessively high performance standards, accompanied by overly critical self-evaluations and concerns regarding others’ evaluations [48]. We considered this personality factor to be relevant to our theoretical rationale, as numerous studies have linked it to elevated perceived stress [49–51], obsessive-compulsive behaviors [52], and excessive behaviors frequently conceptualized as addictive behaviors, such as workaholism [53] or physical exercise dependence [54]. In the multidimensional model provided by Foster et al. [55], perfectionism traits can be viewed as either adaptive (i.e., Personal Standards and Organization) or maladaptive (Concern over Mistakes, Parental Expectations, and Doubts about Actions). In this model, Personal Standards and Organization reflect some of the positive characteristics of perfectionism, especially with respect to planning and completion of tasks, while the other three traits reflect the negative characteristics of perfectionism. In the present study, we expected that Personal Standards and Organization would be protective factors against PSU, as these functional aspects of perfectionism have been positively linked to psychological well-being and life satisfaction [56]. In contrast, we expected that Concern over Mistakes, Parental Expectations, and Doubts about Actions would be risk factors associated with more PSU because an elevated level of parental expectations has been associated with increased perceived stress for students with an Asian cultural background [57], potentially resulting in PSU as a coping process, and Concern over mistakes and Doubts about Actions could promote PSU as a consequence of compulsive checking tendencies [58].

## Methods

### Sample

From the definition of classification for higher education institutions of Mainland China, we performed a stratified cluster random sampling strategy in the city of Changsha, the capital of Hunan Province. This city is one of the largest educational centers in Mainland China, attracting many high school students from all administrative regions for their higher education. All military universities and colleges were excluded from this study because of their particular characteristics and accessibility. Eight universities or colleges, including two important national universities, two provincial colleges, and four vocational colleges, were randomly selected. In these eight schools, 35 classes containing 1205 undergraduates were randomly selected from all of the classes of public courses (e.g., English, ethics, and advanced mathematics) in order to attract students with heterogeneous backgrounds and grades. All the undergraduates in the classes were invited to participate in the study. A total of 1121 undergraduates (93%) filled out the questionnaires, but 51 (4.5%) were excluded from the analysis because they failed to complete the questionnaires in their entirety. Eight more participants were excluded because they were using traditional mobile phones. Therefore, the final sample consisted of 1062 participants (572 females and 490 males) whose ages ranged from 17 to 26 years (M = 20.65 ± 1.54).

### Procedure

This cross-sectional research was conducted between April and May 2015. The protocol was approved by the ethics committee of the Second Xiangya Hospital, Central South University. The distributed survey explicitly stated the purposes of the study and notified the participants that they provided informed consent when they accepted filling out the anonymous survey. Because winter vacation and final examinations might greatly change students’ smartphone use pattern and stress levels, which may eventually influence the results of the study, the entire investigation was conducted in the middle of the semester. The average time for completing the survey was approximately 20 minutes. All the participants received monetary compensation of about 5 RMB (0.8 US dollars) at the end of the survey. To avoid typing errors, we entered information into all questionnaires by using a double-entry strategy in EpiData, version 3.1 (EpiData Association, Odense, Funen, Denmark).

### Measures

#### Socio-demographics

Several items were designed to collect students’ basic socio-demographic data, such as gender, age, grade, hometown, registered permanent residence (urban/rural), family type (single-parent family/non-single-parent family; one-child family/non-one-child family), monthly family income, and science or humanities division in high school (humanities/science/non-division). Monthly income from the family is a main resource to cover living expenses for most Chinese undergraduates. It was measured with the question: “How much money do you get from your family (or your guardians) per month on average?” Responses were recorded in one of three categories: ≤1000 RMB, 1000–1500 RMB, and ≥1500 RMB.

#### Smartphone use feature

Specific information regarding smartphone use and habits were collected, including the age at which the participants had their first mobile phone, the operating system of their smartphone (iOS, Android, other), the top three most-often-used functions (games, e-book reading, Internet surfing, office work, social networking services, video watching, photographing or filming, and other), daily smartphone use time, frequency of changing their mobile phone, and monthly smartphone bill.

#### Problematic Cellular Phone Use Questionnaire (PCPUQ)

The PCPUQ is a self-administered questionnaire comprising 12 dichotomic (yes or no) items. An optimal cutoff point has been developed for this instrument on the basis of the results from a representative Chinese sample in Taiwan by Yen et al. [43]. The PCPUQ was developed according to the taxonomies of substance dependence described in the 4th edition (text revision) of the *Diagnostic and Statistical Manual of Mental Disorders*, and it can be used to determine the occurrence of symptoms of problematic mobile phone use. The questionnaire is subdivided into two parts. The first seven questions ask whether participants had any symptoms suggestive of problematic mobile phone use in the preceding year, whereas the last five questions ascertain participants’ subjective functional impairment caused by excessive mobile phone use. According to Yen et al. [43], the 2-week test-retest reliability (kappa) of the items on the PCPUQ ranged from 0.41 to 0.78, and the kappa between participants’ self-reports and their parents’ reports ranged from 0.26 to 0.44. Cronbach’s alpha was 0.85 in the original study [43], whereas it is 0.72 in the current study. The Chinese version of the 12-item PCPUQ and descriptions of the items are provided in an additional file (see Additional file 1).

Because the PCPUQ was not confined to any specific type of mobile phone, it can also be used for the study on PSU. From the classification provided by Wang et al. [59], participants who had positive responses to four or more of the first seven questions and any of the last five questions were classified as having PSU in the current study.

#### Chinese Frost Multidimensional Perfectionism Scale (CFMPS)

Based on the original English version of the Frost Multidimensional Perfectionism Scale developed by Frost et al. [55] and its Chinese version translated by Cheng et al. [60], Zi and Zhou [61] developed the CFMPS for undergraduates in Mainland China. The CFMPS is a 27-item self-report that measures various facets of perfectionism, namely, Concern over Mistakes (e.g., “If I fail at work/school, I am a failure as a person”), Personal Standards (e.g., “If I do not set the highest standards for myself, I am likely to end up a second-rate”), Parental Expectations (e.g., “My parents wanted me to be the best at everything”), Doubts about Actions (e.g., “Even when I do something very carefully, I often feel that it is not quite right”), and Organization (e.g., “Organization is very important to me”). Confirmatory factor analysis confirmed that the factorial structure of CFMPS is adequate and corresponds to that proposed in the original study (*χ* ^2^/df = 1.44, goodness-of-fit index = 0.89, comparative fit index = 0.93, incremental fit index = 0.93, root mean square error of approximation = 0.043) [61]. In the original study [61], Cronbach’s alpha of each subscale ranged from 0.64 to 0.81, and the 2-week test-retest reliability (kappa) of each subscale ranged from 0.63 to 0.82. In the current study, Cronbach’s alpha of each subscale ranged from 0.71 to 0.84. All items are answered on a 5-point Likert scale ranging from 1 (never) to 5 (always), and all items of each subscale are summed, with a high score indicating high proneness toward perfectionism.

#### Zung Self-Rating Depression Scale (SDS)

The SDS is a 20-item self-reported measure of depression symptoms, originally developed by Zung [62]. Abundant literature has shown that the SDS is a reliable and valid instrument for measuring depressive symptoms [63–66]. The SDS has been translated into Chinese and its validity and reliability have been confirmed in a Chinese sample [67]. Cronbach’s alpha of the Chinese version of SDS was reported to be 0.86 and the test-retest reliability (3-week) was 0.83. Moreover, the correlation coefficient between SAS and the Center for Epidemiological Survey Depression Scale was 0.60 [67]. In our study, Cronbach’s alpha was 0.83. All items are answered on a 4-point Likert scale ranging from 1 (never) to 4 (always). Participants are required to rate each item according to how they felt during the preceding week. The sum of the 20 items produces a raw score reflecting participants’ severity of depressive symptoms.

#### Zung Self-Rating Anxiety Scale (SAS)

The SAS is a 20-item self-reported measure of anxiety symptoms, originally developed by Zung [68]. It is a commonly used screening instrument characterized by elevated validity and reliability [69, 70]. The SAS has been translated into Chinese and the quality of its psychometric properties has been verified in a Chinese sample. Cronbach’s alpha of the Chinese version is 0.93, and the correlation coefficient between SAS and two expert-rated scales was -0.71 (Global Assessment Scale) and 0.81 (severity subscale of Clinical Global Impression Scale) [71]. In the current study, Cronbach’s alpha was 0.81. All items are answered on a 4-point Likert scale ranging from 1 (never) to 4 (always). Participants are required to rate each item according to how they felt during the preceding week. The sum of the 20 items produces a raw score reflecting participants’ severity of anxious symptoms.

#### Perceived Stress Scale (PSS)

The PSS is a self-reported measure composed of 14 items measuring the degree to which an individual perceives aspects of his or her life as being uncontrollable, unpredictable, and overloading [46, 72]. This scale is established as being characterized by adequate psychometric properties [73], and it has been translated and validated in Chinese [74]. The reported Cronbach’s alpha of the Chinese version was 0.78, and its 2-week test-retest reliability (kappa) was 0.78. The factorial structure of the Chinese PSS was also demonstrated as being in accordance with its original version. In the current study, Cronbach’s alpha was 0.77. All items are answered on a 5-point Likert scale ranging from 0 (never) to 4 (very often), indicating how often the participants have felt or thought a certain way within the past month. The sum of all items produces a raw score of perceived stress.

### Statistical analysis

Data analysis was performed with SPSS, version 19.0 (IBM Corp., Armonk, NY, USA). Cronbach’s alpha coefficients were calculated for reliability analysis. Socio-demographic characteristics, smartphone use features, and scale scores were compared between participants with and without PSU by using chi-square tests, Student’s t tests, and Mann-Whitney U tests.

After the univariate analysis, a binary logistic regression analysis was computed to identify predictors of PSU. The independent variables entered in the model (11 variables) were selected a priori based on our theoretical rationale. Odds ratios (ORs) and 95% confidence intervals (CIs) were used to estimate the effect size in the model. A *p* value less than 0.05 was considered statistically significant. All computed tests were two-tailed.

## Results

### Demographic characteristics

The final sample included 1062 undergraduates. Of the total smartphone users, 46.14% were males, were aged 17 to 26 years (M = 20.65 ± 1.54), and were studying in 109 different majors for their bachelor’s degree. Although all participants currently study in Changsha, they originally came from 31 different provincial administrative areas, which covers all areas of Mainland China.

### The prevalence of PSU

On the basis of the aforementioned cutoff point [43, 59], the prevalence of identified PSU was 21.3% (226 participants). Socio-demographic comparisons between the two groups are provided in Table 1.

**Table 1:** Comparisons of socio-demographics between undergraduates with and without PSU

Socio-demographics	Non-PSU: $\overline{\mathrm{X}}$ ±SD; n (%)	PSU: $\overline{\mathrm{X}}$ ±SD; n (%)	Multiple comparisons	Test value: t;*X* ^2^	*p*
Age	20.66 ± 1.575	20.62 ± 1.407		0.39	0.700
Gender				0.067	0.795
Female	452 (54.1)	120 (53.1)			
Male	384 (45.9)	106 (46.9)			
Grade				7.385	0.117
1	178 (21.3)	50 (22.1)			
2	221 (26.4)	74 (32.8)			
3	235 (28.1)	57 (25.2)			
4	167 (20)	42 (18.6)			
5 (medical major only)	35 (4.2)	3 (1.3)			
Residential area				0.204	0.652
Rural	452 (54.1)	126 (55.8)			
Urban	384 (45.9)	100 (44.2)			
Family type 1				0.016	0.899
Single parent	65 (7.8)	17 (7.5)			
Non-single parent	771 (92.2)	209 (92.5)			
Family type 2				0.227	0.634
One child	329 (39.4)	85 (37.6)			
Non-one child	507 (60.6)	141 (62.4)			
Science-humanities division (in high school)			1-2	11.652	0.001
Science (1)	632 (75.8)	145 (64.4)			
Humanities (2)	202 (24.2)	80 (35.6)			
Non-division	2 (-)	1 (-)			
Monthly income from family				11.058	0.004
≤ 1000 RMB (1)	425 (50.8)	122 (54.0)	1–2	4.216	0.040
1000–1500 RMB (2)	321 (38.4)	65 (28.8)	1–3	3.617	0.057
≥ 1500 (3)	90 (10.8)	39 (17.2)	2–3	10.762	0.001
Total	836	226			

*PSU* problematic smartphone use

### Differences of smartphone use features (PSU vs. Non-PSU)

Except for the basic functions traditionally associated with mobile phone use (i.e., calling and Short Message Service), the top three most-often-used functions reported in this study were social networking services (79.66%, *n* = 846), Internet surfing (52.29%, *n* = 562), and video watching (31.45%, *n* = 334). However, there was no significant difference in usage preferences between problematic and non-problematic users regarding these three functions (*χ* ^2^ = 3.383, *p* = 0.184). Comparisons of other smartphone use features between the two groups are provided in Table 2.

**Table 2:** Comparisons of smartphone use features between undergraduates with and without PSU

Smartphone use features	Non-PSU: $\overline{\mathrm{X}}$ ±SD; n (%)	PSU: $\overline{\mathrm{X}}$ ±SD; n(%)	Multiple comparisons	Test value: Z; *X* ^2^	*p*
Age for first mobile phone	16.00 ± 2.286	16.16 ± 2.092	-	0.743	0.458
Number of mobile phones			-	3.896	0.048
1	655 (78.3)	163 (72.1)			
2 or more	181 (21.7)	63 (27.9)			
Daily use time, hours				23.582	<0.001
≤ 2 (1)	226 (27)	41 (19.9)	1–2	0.213	0.644
2–4 (2)	319 (38.2)	64 (27.7)	1–3	17.512	<0.001
≥ 4 (3)	291 (34.8)	121 (52.4)	2–3	17.813	<0.001
Operating system				2.074	0.355
IOS (Apple)	171 (21)	45 (20.1)			
Android (Google)	645 (79)	179 (79.9)			
Other	20	2	-	-	-
Frequency of mobile phone change				19.291	<0.001
Not changed yet	87	21	-	-	-
≤ 1 year (1)	117 (15.6)	56 (27.3)	1–2	8.779	0.003
1–2 years (2)	411 (54.9)	111 (54.2)	1–3	19.083	<0.001
≥ 2 years (3)	221 (29.5)	38 (18.5)	2–3	4.837	0.027
Monthly smartphone bill				13.137	0.001
≤ 50 RMB (1)	424 (50.7)	89 (39.4)	1–2	5.907	0.015
50–100 RMB (2)	363 (43.4)	112 (49.5)	1–3	11.162	0.001
≥ 100 RMB (3)	49 (5.9)	25 (11.1)	2–3	3.560	0.059
Total	836	226			

*PSU* problematic smartphone use

### Comparison of psychological factors (PSU vs. Non-PSU)

With the exception of the Personal Standards (PS) facet of the CFMPS, the risk factors considered in the present study were all significantly different between the two groups (see Table 3).

**Table 3:** Comparisons of seven scale scores between undergraduates with and without PSU

Scale	Non-PSU: $\overline{\mathrm{X}}$ ±SD	PSU: $\overline{\mathrm{X}}$ ±SD	Non-PSU: median (Q25; 75)	PSU: median (Q25; 75)	Test value: Z	*p*
CFMPS	79.11 ± 14.03	85.20 ± 15.27	79 (69; 89)	83 (77; 97)	−5.33	<0.001
CFMPS-CM	11.66 ± 4.82	14.47 ± 5.21	11 (8; 15)	14 (10; 18)	−7.46	<0.001
CFMPS-DA	11.77 ± 3.56	13.97 ± 3.31	12 (9; 14)	14 (12; 16)	−8.07	<0.001
CFMPS-PS	17.39 ± 4.98	17.95 ± 5.02	17.50 (14; 21)	18 (15; 22)	−1.41	0.160
CFMPS-OR	24.35 ± 4.30	23.18 ± 4.67	25 (22; 28)	23 (20; 27)	−3.50	<0.001
CFMPS-PE	13.94 ± 4.61	15.62 ± 4.53	14 (11; 17.75)	16 (13; 19)	−4.73	<0.001
SDS	35.03 ± 7.68	39.38 ± 8.32	34 (29; 40)	40 (34; 46)	−7.15	<0.001
SAS	33.34 ± 6.61	37.69 ± 8.76	32 (29; 37)	37 (30.75; 44)	−6.67	<0.001
PSS-10	23.62 ± 5.63	27.36 ± 5.48	24 (20; 27)	28 (25; 30)	−6.78	<0.001

*PSU* problematic smartphone use, *CFMPS* Chinese Frost Multidimensional Perfectionism Scale; *CFMPS*-*CM* CFMPS-Concern over Mistakes subscale, *CFMPS*-*DA* CFMPS-Doubts about Actions subscale, *CFMPS*-*PS* CFMPS-Personal Standards subscale, *CFMPS*-*OR* CFMPS-Organization subscale, *CFMPS*-*PE* CFMPS-Parental Expectations subscale, *SDS* Zung Self-Rating Depression Scale, *SAS* Zung Self-Rating Anxiety Scale, *PSS*-*10* Perceived Stress Scale-10 item

### Prediction of PSU

From the results of the univariate analysis, a logistic regression analysis was then performed to predict PSU. To avoid multicollinearity-related issues, we collapsed the variables anxiety and depression into a new variable of “emotional symptoms” by adding the scores of the SDS and the SAS. Gender, grade, science-humanities division, monthly income from the family, and all psychological risk factors, including emotional symptoms, were then included as independent predictors. Inspection of residuals and multicollinearity effects showed that the conditions of application for regression analysis were respected (variance inflation factor: 1.03–2.09). Eleven predictors were entered in the final model and six of these predictors were statistically significant (see Table 4). Together, these variables predict 22.2% of PSU in the undergraduates.

**Table 4:** Results of regression analysis with PSU as a criterion variable^a^ (*n* = 1062)

	Crude		Adjusted		
Predictors	OR	95% CI	OR	95% CI	*p*
Gender: 0 = male; 1 = female	0.96	0.72–1.29	0.80	0.55–1.17	0.251
Grade	0.89	0.78–1.01	0.89	0.77–1.04	0.135
Science-humanities division: 0 = Science; 1 = Humanities	1.73	1.26–2.37	2.14	1.45–3.16	<0.001
Monthly income from family (RMB)			-	-	0.003
0:1000–1500; 1: ≤1000	1.42	1.02–1.98	1.34	0.93–1.93	0.112
0:1000–1500; 1: ≥1500	2.14	1.35–3.39	2.45	1.46–4.13	0.001
Emotional symptoms (SAS + SDS)	1.04	1.03–1.05	1.01	1.01–1.03	0.039
PSS	1.14	1.10–1.17	1.06	1.02–1.10	0.003
CFMPS-DA	1.20	1.15–1.26	1.15	1.08–1.22	<0.001
CFMPS-PE	1.09	1.05–1.12	1.04	1.00–1.08	0.047
CFMPS-CM	1.11	1.08–1.14	1.04	0.98–1.09	0.068
CFMPS-OR	0.94	0.91–0.98	0.96	0.92–1.01	0.089
CFMPS-PS	1.02	0.99–1.05	0.96	0.92–1.01	0.085
(Intercept)	-	-	0.006	-	<0.001

*PSU* problematic smartphone use, *OR* odds ratio, *CI* confidence interval, *SAS* Zung Self-Rating Anxiety Scale, *SDS* Zung Self-Rating Depression Scale, *PSS* Perceived Stress Scale, *CFMPS*-*DA* Chinese Frost Multidimensional Perfectionism Scale-Doubts about Actions subscale, *CFMPS*-*PE* CFMPS-Parental Expectations subscale, *CFMPS*-*CM* CFMPS- Concern over Mistakes subscale, *CFMPS*-*OR* CFMPS-Organization subscale, *CFMPS*-*PS* CFMPS- Personal Standards subscale

^a^Criterion variable setting: 0 = non-PSU; 1 = PSU

## Discussion

Previous studies indicated that PSU might be important in Chinese undergraduates and thus constitutes a public health issue in Mainland China. Nevertheless, data about epidemiology and risk factors of PSU in Mainland China are scarce and most often not published in international scientific outlets. Our study aimed to fill an important gap in the literature by investigating the prevalence and correlates of PSU in Mainland China, relying on a large-scale cross-sectional study conducted in a representative sample of Chinese undergraduate students. The current study also provides an important empirical predictive model of PSU on the basis of the stress-coping theoretical framework. Identified predictors of PSU included majoring in the humanities, elevated monthly family income, elevated emotional symptoms, high perceived stress, and perfectionism-related factors (high doubts about actions, high parental expectations).

### The prevalence and use features of PSU

In this study, 99.2% of the Chinese undergraduates were smartphone users, and the prevalence of PSU was estimated to be 21.3%. The undergraduates with PSU tended to own more mobile phones (two or more), acquire new mobile phones more often (<1 year), and spend more time (>4 h per day) and money (>50 RMB per month) on smartphone use.

The prevalence of PSU in the current study was higher than that in previous studies conducted in neighboring regions (e.g., Taiwan and South Korea). Measured with the same psychometric instrument, the prevalence of problematic mobile phone use was indeed estimated to be 16.4% to 16.7% in the population of adolescents of Taiwan [8, 43]. This is all the more striking, as we used a stricter definition of the condition in the current study: Endorsing at least one index of functional impairment was required to be classified as displaying PSU. Indeed, the prevalence of PSU would have become 36.3% if the functional impairment criterion had been ignored. For example, based on the same instruments and diagnosis criteria, the prevalence of PSU in Taiwan adolescents can be estimated to be 10.54% [59]. According to the survey of PSU completed by the National Information Society Agency of South Korea in 2012, the prevalence of PSU among Korean youth (11.4%) was much lower as well [75]. In addition, it was recently reported that the prevalence of problematic mobile phone use among Chinese adolescents was estimated to be 26.2% in 2012 [18]. It is worth noting, however, that the screening questionnaire used in the current study (PCPUQ) was developed by directly transposing substance abuse criteria to the diagnosis of PSU, an approach that has been criticized and is susceptible to artificially inflated prevalence rates [7]. Yet, the high prevalence rate identified in the current study calls for considering PSU to be a potential public health concern in Mainland China.

### Socio-demographics

The science-humanities division was identified as a suitable predictor for PSU in our final model (OR = 2.14, *p* < 0.001). In the context of higher education in China, the science-humanities division of high school education is a unique classification system for senior high school students, who have to decide whether they will choose to study mainly humanities or science at an early age (usually at the age of 16). Similar to a previous study conducted by Abu-Jedy [30], our study also revealed that compared with the undergraduates who mainly accepted education in science at high school, those who mostly accepted education in the humanities were more likely to use smartphones problematically, which suggests that undergraduates majoring in humanities in high school are more at risk for PSU. Since it is reported that college students majoring in humanities have a lower level of psychological well-being than do those majoring in science [76] and since poor well-being is a risk factor for addictive behaviors [77], it is possible that the lower level of psychological well-being could potentially make humanities students more vulnerable to PSU. Furthermore, previous studies also showed that there are different behavioral tendencies (such as different problem-solving strategies) between arts students and science students [78, 79], which is another possible explanation for the difference.

We also found that monthly income from the family had an impact on smartphone use. For most Chinese undergraduates, monthly income from the family is their only source of income and thus directly affects their lifestyle and daily behaviors. Although previous studies revealed mixed associations between family income and PSU [26, 27], the current study found elevated family income (≥1500 RMB) to be a risk factor for PSU (OR = 2.45, *p* = 0.001). It thus appears that undergraduates who are less constrained by finances are more prone to use their (smart)phone excessively, which may ultimately result in addiction symptoms.

No gender difference was identified regarding PSU in this study, which is in accordance with preliminary evidence [16, 26, 44]. Yet, some studies reported that females are more addicted than males to their mobile phone. Nevertheless, this was particularly the case in earlier studies that focused on the first generations of mobile phones [22–25], and the most significant differences were associated with specific mobile phone functions, especially text message use [23, 24]. It is thus possible that the numerous functions of the latest generations of smartphones promote excessive use in both males (e.g., video games) and females (e.g., social networking).

### Emotional symptoms, perceived stress, and perfectionism

Previous studies reported that anxiety and depression were both associated with PSU [5, 42, 44]. In our study, the severity of emotional symptoms (anxiety and depression) was identified as a suitable predictor. In fact, consistent evidence has shown that anxiety and depression are associated with various kinds of addictive behaviors, including both substance abuse and behavioral addiction [39, 41, 80–82], which suggests common risk pathways between these two types of emotional disorders and addictive behaviors. In line with our assumptions, it is possible that PSU serves as a way to escape from feelings of anxiety or depression, which could produce a relieving effect, thus favoring the onset of addictive patterns of use [42, 45]. Moreover, it has been reported that addiction symptoms may promote retroactive emotional symptoms [81, 83, 84], which might ultimately result in a vicious cycle that reinforces the addictive behavior. Our findings are also in accordance with recent proposals that PSU might be displayed by anxious or insecure individuals who need reassurance by significant others [4].

Perceived stress was also shown to be a predictor for PSU in our study. In line with this finding, recent studies found a positive association between perceived stress and PSU [6, 47]. Similarly, Internet-related disorders were also reported to be positively associated with anxiety and stressors stemming from interpersonal and school-related problems [45]. In addition, Leung [85] found that young people tended to increase their Internet use to manage moods, compensate for social interaction, and escape from reality when they were faced with excessive life stress. On the basis of these results, PSU can be conceptualized as a maladaptive coping process displayed to face stressful contexts or events. Moreover, similar to the potential reverse causality between emotional symptoms and PSU, the association between stress and PSU perhaps reflects a reinforcing spiral, rather than a unidirectional pathway.

To the best of our knowledge, the current study is the first to relate PSU to perfectionism. By using a multidimensional model in which specific perfectionism traits can be viewed as either adaptive or maladaptive [55], our study revealed that when all potential confounding factors are taken into account, PSU is positively predicted by a proneness to doubts about actions and heightened parental expectations. First, *Doubts about actions* has been proposed as a core feature of perfectionism that is underlain by inflexible standards and low self-confidence and associated with compulsive checking and procrastination [55, 86, 87]. Accordingly, it can be hypothesized that for these perfectionists, PSU is the consequence of uncontrolled compulsions (e.g., constant checking of mobile phone apps) and/or procrastination (e.g., using mobile phone apps to avoid being faced with a risk of failure). Second, elevated *Parental expectations* has been related to increased perceived stress and emotional symptoms [55], and maltreated youths who have been faced with socially prescribed perfectionism tend to display maladaptive coping strategies [88]. Adolescents faced with such a parental educational style are thus more prone to display excessive or addictive behaviors to relieve negative affect states, which may (among other things) result in PSU. In a recent study, Roser et al. [89] found that a low educational level of the parents was significantly associated with problematic mobile phone use, which also underscores the potential role of parental education in the development and maintenance of problematic mobile phone use.

### Limitations

Several limitations of this study should be acknowledged. First, the cross-sectional design hindered any causal interpretation of the relationships shown between PSU and other variables. Second, the study was conducted in only one educational center of China, which may limit the generalizability of the findings. Large-scale studies with national sample populations are thus highly recommended in the future. Third, data collection was based only on self-reports; therefore, the findings are potentially influenced by response bias or errors. Finally, it is worth noting that perfectionism traits have been related to reduced self-esteem [90], a factor not assessed in the current study but one that has been consistently linked to problematic mobile phone use in previous studies [8, 9, 16]. Accordingly, further studies performed to disentangle the relationships between perfectionism and PSU should control for the potential confounding effect of self-esteem.

## Conclusions

In this study, we estimated the prevalence of PSU among Chinese undergraduates to be 21.3%, which suggests that PSU has become a public health issue in Mainland China. On the basis of the stress-coping theory, we also provided a predictive model that identified several socio-demographic and psychological factors as being involved in PSU. The findings of the current study, obtained in a random and thus representative sample of undergraduates, opens up new avenues in terms of prevention and regulation policies. For example, it could be useful to develop prevention actions that aim to explain how mobile phone use can become problematic if used to escape real-life problems or to reduce negative affect. At the same time, a more systematic promotion of adaptive coping strategies (e.g., problem solving-based coping strategies) in schools and universities could help to reduce anxiety and stress and thus eventually reduce PSU in students.

## Additional file

Additional file 1: Provides the Chinese version of the 12-item PCPUQ (**Table S1**) and English descriptions of each item (**Table S2**). (DOCX 15 kb)

## Abbreviations

CFMPS: Chinese Frost Multidimensional Perfectionism Scale
PCPUQ: Problematic Cellular Phone Use Questionnaire
PSS: Perceived Stress Scale
PSU: Problematic smartphone use
SAS: Zung Self-Rating Anxiety Scale
SDS: Zung Self-Rating Depression Scale
