# Supplementary material for: Prevalence and correlates of problematic smartphone use in a large random sample of Chinese undergraduates
Source: BMC Psychiatry. 2016 Nov 17;16:408. doi: 10.1186/s12888-016-1083-3 (PMC5114822; doi:10.1186/s12888-016-1083-3)
Supplement: Additional file 1: — Provides the Chinese version of the 12-item PCPUQ (Table S1) and English descriptions of each item (Table S2). (DOCX 15 kb) [file 12888_2016_1083_MOESM1_ESM.docx]

**The Problematic Cellular Phone Use Questionnaire (PCPUQ)**

Table S1. Chinese version of the 12-item PCPUQ (Yen et al., 2009)

| **指导语：**请您仔细阅读下列条目，并根据您智能**手机使用**的实际情况，做出“是”或“否”的回答。 | | |
| --- | --- | --- |
| 1.和开始使用手机时相比，现在每天使用手机的时间明显增加了。 |  |  |
| 2.如果突然没有手机，或突然被限制使用手机，会觉得难受。 |  |  |
| 3.在手机使用上所花的时间或金钱常常超过了自己的预定程度。 |  |  |
| 4.曾经尝试少花一定的时间或金钱在手机上，却没有成功。 |  |  |
| 5.明明知道已经花太多时间或太多钱在手机上，却无法控制。 |  |  |
| 6.因使用手机而放弃和朋友或家人相处的机会，或停止原本喜欢的休闲活动。 |  |  |
| 7.明知自己的身心健康问题可能与手机过度使用有关，却仍不愿改变。 |  |  |
| 8.使用手机已经影响您的学业成绩。 |  |  |
| 9.使用手机已经影响和您和同学、朋友的关系。 |  |  |
| 10.使用手机已经影响和您和父母、兄弟姊妹的关系。 |  |  |
| 11.使用手机已经影响您的身体或心理健康。 |  |  |
| 12.为了使用手机已经让您惹上任何经济或法律纠纷（如拿别人手机、欠缴手机费用、购买赃物等）。 |  |  |

Table S2. Descriptions of the twelve items within the PCPUQ

| ***Symptoms of problematic cellular phone use (CPU)*** |
| --- |
| 1. Tolerance: a marked increase in the frequency and duration of CPU needed to achieve satisfaction |
| 2. Withdrawal symptoms without CPU |
| 3. CPU for a period of time longer or more frequent than intended |
| 4. Persistent desire and/or unsuccessful attempts to cut down or reduce CPU |
| 5. Excessive time spent on CPU or excessive effort spent on activities necessary to obtain cellular phone |
| 6. Giving up or reducing important social, academic, or recreational activities because of CPU |
| 7. Continued heavy CPU despite knowledge of having a persistent or recurrent physical or psychological  problem likely to have been caused or exacerbated by CPU |
| ***Functional impairment caused by CPU*** |
| 8. Poor relationship with friends or classmates |
| 9. Poor academic performance |
| 10. Poor relationship with family members |
| 11. Compromised physical or psychological function |
| 12. Problems in financial affairs |
